# Neoepitopes in Type 1 Diabetes: Etiological Insights, Biomarkers and Therapeutic Targets

**DOI:** 10.3389/fimmu.2021.667989

**Published:** 2021-04-19

**Authors:** Teresa Rodriguez-Calvo, James D. Johnson, Lut Overbergh, Jessica L. Dunne

**Affiliations:** ^1^ Institute of Diabetes Research, Helmholtz Zentrum Muenchen, German Research Center for Environmental Health, Munich, Germany; ^2^ Diabetes Research Group, Department of Cellular and Physiological Sciences, Life Sciences Institute, University of British Columbia, Vancouver, BC, Canada; ^3^ Laboratory Clinical and Experimental Endocrinology, KU Leuven, Leuven, Belgium; ^4^ Janssen Research and Development, LLC, Raritan, NJ, United States

**Keywords:** type 1 diabetes, neoepitopes, autoantigens, pathogenesis, beta-cell, biomarker

## Abstract

The mechanisms underlying type 1 diabetes (T1D) pathogenesis remain largely unknown. While autoantibodies to pancreatic beta-cell antigens are often the first biological response and thereby a useful biomarker for identifying individuals in early stages of T1D, their role in T1D pathogenesis is not well understood. Recognition of these antigenic targets by autoreactive T-cells plays a pathological role in T1D development. Recently, several beta-cell neoantigens have been described, indicating that both neoantigens and known T1D antigens escape central or peripheral tolerance. Several questions regarding the mechanisms by which tolerance is broken in T1D remain unanswered. Further delineating the timing and nature of antigenic responses could allow their use as biomarkers to improve staging, as targets for therapeutic intervention, and lead to a better understanding of the mechanisms leading to loss of tolerance. Multiple factors that contribute to cellular stress may result in the generation of beta-cell derived neoepitopes and contribute to autoimmunity. Understanding the cellular mechanisms that induce beta-cells to produce neoantigens has direct implications on development of therapies to intercept T1D disease progression. In this perspective, we will discuss evidence for the role of neoantigens in the pathogenesis of T1D, including antigenic responses and cellular mechanisms. We will additionally discuss the pathways leading to neoepitope formation and the cross talk between the immune system and the beta-cells in this regard. Ultimately, delineating the timing of neoepitope generation in T1D pathogenesis will determine their role as biomarkers as well as therapeutic targets.

## The Evolving Understanding of the Pathogenesis of Human T1D

The immune system plays a pivotal role in type 1 diabetes (T1D), as demonstrated by numerous studies and experiments *in vitro*, *ex vivo*, in animal models, and in humans (1, 2). However, our understanding of the disease continues to evolve, with a greater recent appreciation for the role of pancreatic beta-cell factors and beta-cells themselves in disease etiology (3, 4). Our knowledge of the immunopathology of T1D is incomplete, partially due to the difficult access to human pancreas samples. This limitation has been partially overcome in recent years thanks to the emergence of several large tissue biobanks like the Network for Pancreatic Organ Donors with Diabetes (nPOD) (5, 6) and the Exeter Archival Diabetes Biobank (EADB) (7), which now permit the investigation of immune cell populations in the human pancreas. The presence of immune cells in the islets, known as insulitis, is a hallmark of T1D (8–11). The importance of CD8+ T-cells in T1D is evident by their abundance in islets that have remaining beta-cells, as well as in those with only a few beta-cells left. CD8+ T-cells are also found in the exocrine portion of the pancreas in individuals with T1D, even when beta-cells are lost (12). While CD4+ T-cells are also present in the islets, they are not considered a major component of the immune infiltrate in established diabetes; this is not unexpected given the more prominent role of CD4+ T-cells in disease initiation (13) rather than in disease progression/amplification. In addition, their potential role in sustaining the effector functions of CD8+ T-cells should not be neglected (14). Both CD4+ and CD8+ T-cell populations decline with beta-cell loss, suggesting that their presence is driven by a beta-cell antigen (11, 12, 15). However, the exact role infiltrating T-cells play in the pathogenesis of the disease remains to be determined both in terms of specificity and function.

Antigen-specific CD8+ T-cells recognizing diverse islet antigens have been detected in the pancreas of individuals with T1D (16). T-cells with single specificity were detected early in the disease process, whereas in long-standing donors, islets usually contained multiple islet-reactive specificities indicating epitope spreading (16). Interestingly, in recent onset cases, different islets harbored different reactivities, which could reflect different stages of the autoimmune process. More recently, a high proportion of preproinsulin (PPI) specific cells have been detected in the islets of donors with T1D (17, 18), confirming previous data obtained from blood samples and highlighting the role of PPI as one of the most prominent antigens in disease pathogenesis (15, 19–22). Attempts to detect neoantigens *in situ* have not been reported so far but are on the horizon. Characterizing the frequency and localization of neoantigens at different disease stages therefore remains an important goal.

## The Expanding Catalogue of Epitopes With Putative Roles in T1D

The exact mechanisms and timing of the antigenic events, the initial loss of tolerance, as well as the role of autoimmunity to both native and modified epitopes in the pathogenesis of T1D remains unknown. Recently, a comprehensive overview of the known T1D epitopes and neoepitopes was published (22). Sixteen CD8+ T-cell conventional epitopes have been identified and five of these are contained within the major known antigens insulin, glutamate decarboxylase (GAD), insulinoma-associated antigen 2 (I-A2), zinc-transporter 8 (ZnT8) and islet-specific glucose-6-phosphatase catalytic subunit-related protein (IGRP). CD8+ T-cells reactive against epitopes from islet amyloid polypeptide (IAPP), insulin gene enhancer protein (ISL1), urocortin-3 (UCN3) and SLC30A8 (also known as ZnT8) have been also identified in the pancreas (23, 24). Their frequencies were higher in T1D donors compared to non-diabetic donors and their phenotype was predominantly antigen-experienced. In addition, CD8+ T-cell reactivity against other granule proteins such as prohormone convertase 2 (PCSK2), secretogranin 3 (SCG3) and 5 (SCG5) have been recently reported, highlighting the immunogenic potential of beta-cell granule proteins (25). The specificity of islet infiltrating T-cells directly sorted or grown from individual islets isolated from donors with T1D has been also investigated (26). Several CD4+ T-cell clones were obtained, which reacted against proinsulin, GAD65 and chromogranin A (ChgA). CD8+ T-cell clones recognized epitopes from insulin, IA-2 and IGRP. The differential antigenic drivers, including number of targets, diversity of epitopes, environmental triggers, host genetics and even clinical age, may contribute to these varying antigen recognition and disease pathways (27).

## Generation of Neoepitopes Through Post-Translational Modifications

In the case of T1D, autoantibodies to native proteins are highly prognostic of future disease but there is little evidence of their pathogenic role (28–30). While loss of tolerance to insulin has long been thought to be involved early in disease pathogenesis, responses to modified proteins may add to disease heterogeneity, in terms of variations in risk and rate of progression to clinical T1D (31). However, clinical evidence has shown that not all cases of T1D start with reactivity to insulin, and reactivities to different non-conventional antigens may be due to different clinical and pathogenic features (32).

**Figure 1:**
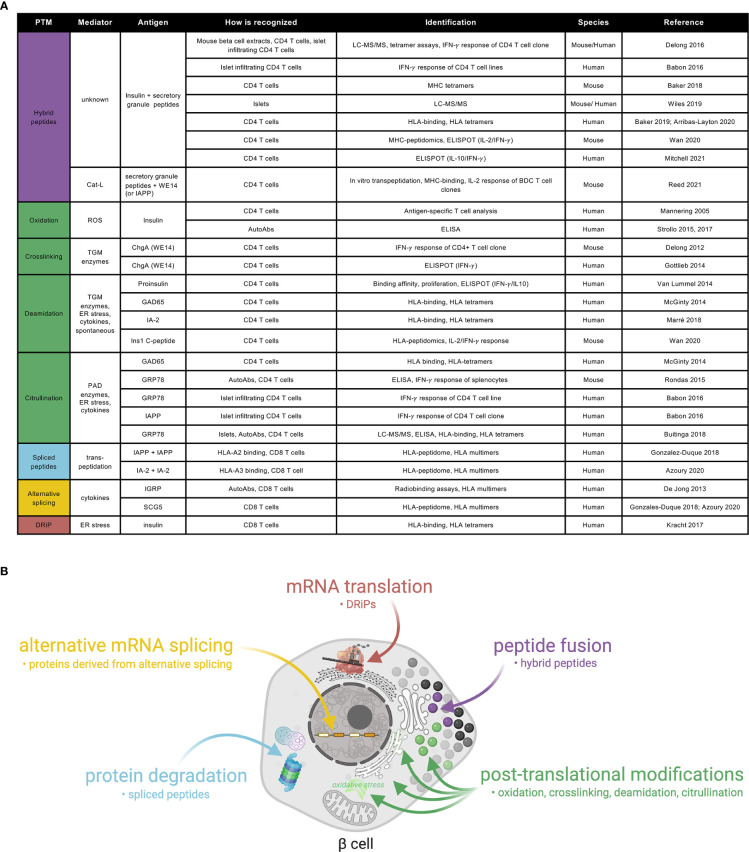
A catalogue of neoepitopes and their production sites/mechanisms in beta-cells. **(A)** Referenced list of known neoepitopes in type 1 diabetes where the type of PTM, the potential mediator, the antigen and how it is recognized and identified, the species in which it was identified and the reference to the original publication are shown. Cat-L, cathepsin L; ROS, reactive oxygen species; AutoAbs, autoantibodies; TGM, tissue transglutaminase; ChgA, chromogranin A; ER, endoplasmic reticulum; PAD, peptidylarginine deiminase; GAD: glutamate decarboxylase; IA-2: insulinoma-associated antigen 2; ZnT8, zinc-transporter 8; IGRP, islet-specific glucose-6-phosphatase catalytic subunit-related protein; GRP78, glucose-regulated protein 78; IAPP, islet amyloid polypeptide; SCG5, secretogranin 5. **(B)** Schematic representation of a beta-cell showing the current view on sub-cellular origin of specific classes of neoepitopes, as well as the types of products produced.

PTMs are part of normal physiological processes. However, they can also be formed as a result of an inflammatory assault restricted to inflamed tissues, and as such, be associated with autoimmunity. As neoepitopes are exclusively expressed in the peripheral target tissue, and not present in their modified form in the thymus, their escape from thymic deletion through negative selection in medullary thymic epithelial (mTEC) cells accounts for the lack of central tolerance to PTMs (33, 34). In the context of T1D, several neoantigens generated through PTMs have been described (**Figure 1**). In some cases, autoantibodies against modified proteins can be both pathogenic and predictors of disease onset (35). Antibodies directed against citrullinated proteins can predate clinical onset of rheumatoid arthritis (RA) by up to two decades (36). The most extensively studied enzymatically mediated PTMs are citrullination, the conversion of arginine into citrulline residues, and deamidation, the conversion of glutamine into glutamic acid residues. These PTMs are mediated by peptidylarginine deiminase (PAD) and tissue transglutaminase (TGM) enzymes, respectively. Citrullinated proteins are preferentially bound by RA-susceptible HLA class II and presented to T-cells, implicating them in disease pathogenesis (37). With similar HLA-susceptible haplotypes shared between RA and T1D, citrullination of multiple proteins has also been implicated in T1D pathogenesis (38, 39). Autoantibodies and circulating and islet infiltrating CD4+ T-cells have been found to react against citrullinated glucose-regulated protein 78 (GRP78) epitopes in T1D individuals (38, 40), following up on earlier observations in NOD mice (41). In addition, circulating CD4+ T-cells reactive against citrullinated GAD65 (39) and islet CD4+ T-cells reactive against citrullinated IAPP have been detected in people with T1D (40). Deamidated peptides, in which glutamine was converted into glutamic acid, were described in both murine T1D (42) and human T1D (39, 43), with several autoantigens identified (39, 42–44).

Oxidative post-translational modifications and cross-linking of proteins are important mechanisms that may contribute to autoreactivity in T1D (**Figure 1**). TGM-mediated crosslinking of the naturally processed ChgA cleavage product WE14 increased its immunogenicity, eliciting strong CD4 T-cell responses both in NOD mice (45) and T1D patients (46). Oxidation of the insulin A-chain, resulting in a disulfide bond formation between two adjacent cysteines, was shown to be responsible for recognition of the peptide by a T-cell clone isolated from the circulation of a T1D patient (47). More recently, autoantibodies against oxidized insulin were detected in prediabetic children (48) and in recently diagnosed T1D subjects (49).

Hybrid insulin peptides (HIPs) are also potential neoantigens, formed through a covalent cross-linking reaction between the C-terminal carboxylic acid group of proinsulin fragments and the N-terminal amine group of other secretory granule peptides (26). HIPs have been identified by LC-MS/MS in human (50) and mouse islets (51), as well as in the mouse MHC-peptidome (42). An increasing number of studies have shown autoreactive responses against such HIPs, both in NOD mice and in human T1D (26, 52). CD4+ T-cells isolated from pancreatic islets recognized different proinsulin C-peptide fragments fused to IAPP1, IAPP2, neuropeptide-Y or insulin A chain (40, 51). Increased reactivity to several HIPs was shown in peripheral blood of T1D patients (53, 54), Furthermore, in genetically at risk individuals, HIPs were detectable, and were shown to have a predominantly pro-inflammatory profile in those that progressed to developing disease (55), making them interesting candidates for novel biomarkers. Finally, a recent report suggests that transpeptidation of beta-cell antigens, mediated by cathepsin L, generates chimeric epitopes through fusion of secretory granule proteins with WE14, for diabetogenic CD4 T-cells (56). In regard to HLA class I epitopes, spliced peptides, generated in the proteasome through a process referred to as transpeptidation by which two different regions of a protein or of two different proteins are fused (57, 58), have been identified in a human beta-cell line by HLA-peptidomics (21, 25) and were recognized by circulating and pancreas-infiltrating CD8+ T-cells from T1D donors. Furthermore, defective ribosomal products (DRIPs) (24) are also regarded as potential neoepitopes against which CD8+ T-cells were shown to be reactive in human subjects.

## Beta-cell Stress as a Contributor to Neoepitope Generation

There are many factors that could theoretically lead to the production of and reactivity to autoantigens in a subset of beta-cells (**Figure 1**). Beta-cells could produce a modified protein *via* genetic or epigenetic up-regulation of transcription, or increased translation. Alternatively, errors in mRNA transcription, splicing, or translation processes could generate neoantigens from modified proteins (24, 59), and they could build up as a result of impaired quality control of the modified protein in the endoplasmic reticulum (ER), post-ER compartments, or during proteosomal degradation (**Figure 1**). Autoantigens may also result from increased expression or activity of PTM modifying enzymes (44). In the case of insulin, the T1D at-risk allele at rs3842753 (60–62) was reported to increase insulin production in a small number of human pancreata (60, 63, 64), a result supported by recent meta-analysis of single-cell RNA sequencing data (65). One might speculate that increased insulin production could both increase neoepitope production and beta-cell vulnerability to ER stress (66). This may be a contributing factor to the increased risk of T1D development observed with childhood obesity (67).

Increasing evidence points to the beta-cell itself as an active player in mediating such processes, thereby participating in its own destruction (3). Beta-cells have a highly developed ER, making it possible for individual cells to react rapidly to changes in metabolic demand and produce enormous amounts of insulin in a short time. On the other hand, this highly specialized secretory task makes the most active beta-cells highly vulnerable to ER stress, which is present to some degree even in normal basal conditions (66). When the demand for protein synthesis and folding overwhelms the capacity of the ER, the cell-autonomous Unfolded Protein Response (UPR) is initiated, aiming to restore ER homeostasis (68). This UPR response is mediated by three UPR ‘sensors’, which are inactive in physiological conditions through their association with the abundant ER chaperone GRP78. With an increased load of unfolded proteins in the ER, GRP78 is released from these UPR sensors, thereby initiating the UPR. When an excessive level of stress is maintained, the UPR fails, and beta-cell apoptosis is triggered (69, 70). ER stress has also been implicated in the generation of neoantigens (71), with increasing recognition of autoreactive T-cell clones specific for deamidated peptides in multiple experimental systems (44, 72). Of importance, such increased immunogenicity was observed when beta-cells were stressed with thapsigargin, but not with tunicamycin, an ER stressor acting through the blocking of glycoprotein synthesis. A similar increase in activity of TGM2 upon inflammatory stress with cytokines has been observed in rodent MIN6 cells and was associated with an increased number of deamidated peptides. Moreover, an increase in non-enzymatically mediated deamidations was observed in this model upon cytokine exposure (73). Finally, insulin DRIP polypeptides increase by Ca-2+ -mediated ER stress, shown by exposure of INS-DRiP-GFP transfected cells to thapsigargin (24).

As to the role of alternative splicing, it has been shown that the beta-cell alternative spliced repertoire is largely affected by the pro-inflammatory cytokines, interleukin-1β (IL1β) and interferon-*γ* (IFN*γ*), changing the expression of more than 30 RNA binding proteins, thereby affecting the splicing of more than 3000 genes involved in beta-cell function and survival (3, 59). In addition, PTMs, alternative splicing and first exon usage are induced by interferon-1α (IFNα) (74). The fact that such transcriptional regulation could lead to neoantigen formation was shown for IGRP, with generation of autoantibodies and CD8 T-cells against a pancreas specific IGRP alternative spliced form (75). Recent evidence further showed that the ‘alternative splicing signature’ is also seen in the immunopeptidome of HLA-A2 and HLA-A3 restricted epitopes, leading to the generation of islet-reactive CD8+ T-cells both in T1D patients and healthy subjects (21, 25). All these studies point to a role for ER stress in increasing the prevalence of a variety PTMs where pro-inflammatory cytokines are likely to expand the repertoire of proteins and transcripts generated by beta-cells.

## Environmental Factors in the Generation of Neoautoantigens

Viruses and other environmental triggers have been implicated in T1D and may not only contribute to beta-cell stress and production of neoepitopes, but may be responsible for antigen-specific targeting. One such potential pathway is molecular mimicry, where viruses, microbiota or other environmental targets express epitopes similar to those expressed on beta-cells. Cross-reactive T-cells against these epitopes have the potential to eliminate both the environmental stimulus (i.e. viruses) and pancreatic beta-cells (76–78). Cross-reactivity between epitopes present in Coxsackievirus B (CVB) and GAD65 have been reported (79, 80). Furthermore, a dominant epitope present in IA-2 elicited T-cell responses in relatives and shared sequence similarity with a protein of human rotavirus (81). This IA-2 epitope also had some identity and similarity to sequences in Dengue, cytomegalovirus, measles, hepatitis C, and canine distemper viruses, and the bacterium *Haemophilus influenzae*. Interestingly, two other IA-2 epitopes were similar to amino acid sequences in milk, wheat, and bean proteins (81). More recently, ZnT8-reactive CD8+ T-cell clonotypes were found to cross-recognize a *Bacteroides stercoris* mimotope (23). Based on this evidence, environmental factors have the potential to elicit autoreactive immune responses. In this scenario, environmental cues could be a requirement or a contributing factor for neoepitope formation in the pancreas.

## Does Neoepitope Generation Lead to Autoimmunity and Beta-Cell Killing in Type 1 Diabetes?

The identification and timing of T-cells reactive against antigens in the pancreas remains a challenging task. Further studies are needed, as illustrated by the apparent lack of correlation between the presence of antigen-specific cells in the periphery and in the pancreas (23). Given that most cells present in the islets have unknown reactivities, it becomes clear that we might be only looking at the tip of the autoreactive iceberg. Models indicate that only 1-2% of antigen-specific cells are enough to achieve effective killing (82). On the other hand, if this is the case, why does it take so long for T-cells to kill their target? It could be that T-cell access to islets is asynchronous due to the expression of different antigens in different islets, at different times. Or perhaps T-cells are not attracted to a given islet unless there is inflammation or a triggering event locally, one that might be able to generate neoantigens. Furthermore, there is little clinical evidence to suggest whether these processes are taking place before disease onset, at a later stage of the disease, or both. A recent longitudinal study on individuals at-risk indicate the presence of neoepitopes in early stages of T1D. Individuals progressing to T1D showed a predominant pro-inflammatory T-cell reactivity against few of the HIPs analyzed (55).

It is clear that individuals carrying certain HLAs are prone to autoreactivity. In this context, it is tempting to speculate that the fragility of beta-cells and their susceptibility to environmental insults and conditions of incremental stress are likely to further unbalance an already compromised genetic system. Considering that neoepitopes are likely to arise before or during disease development, we envision a scenario in which beta-cells themselves actively contribute to neoepitope formation. The upregulation of HLA-I in the islets prior to clinical diagnosis is a good indicator of the potential capacity of beta-cells to present self-epitopes to the immune system, a phenomenon that is not well understood, but that many are actively investigating.

In this regard, and closing the speculative circle, we hypothesize that an environmental insult (i.e. viral infection/s) could induce an anti-viral response and the local production of cytokines (i.e. type I interferons). Anti-viral response molecules can induce the upregulation of HLA-I. At the same time, viruses could induce a translational arrest in beta-cells, hampering insulin production. Also, other forms of environmental stress, such as chemicals, dietary components may cause an initial trigger leading to ER or oxidative stress.

**Figure 2:**
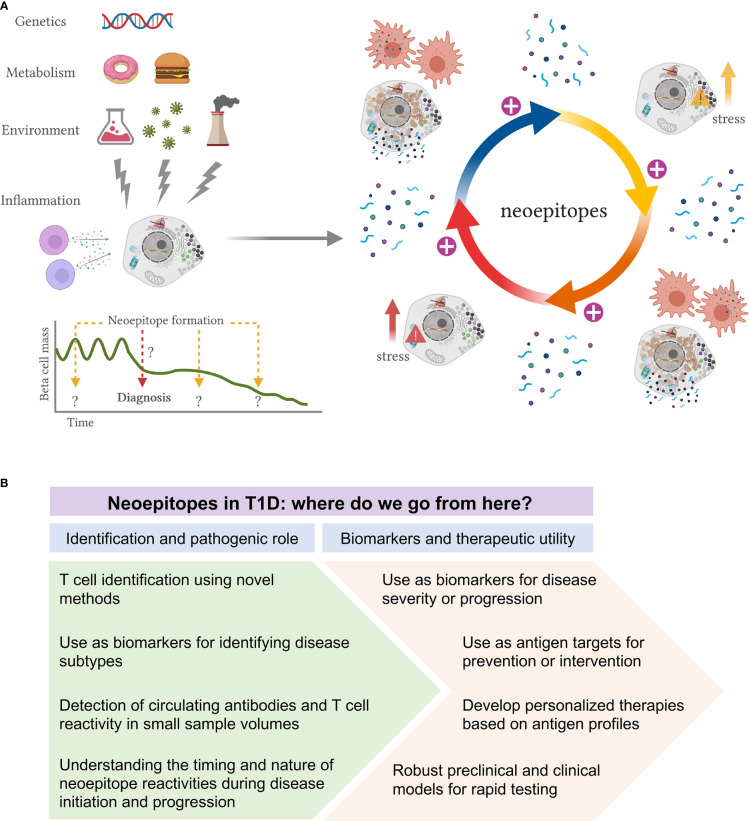
Proposed model of neoepitope generation and roadmap to their identification and utility in T1D. **(A)** Schematic representation of how neoepitope generation could lead to autoimmunity and beta-cell killing in T1D. Genetic predisposition, metabolism, environmental insults and immune inflammation are some of the factors that may lead to the formation of a first wave of non-conventional proteins. Impaired clearance of such stressed or dying beta-cells expressing modified proteins will cause activation of APCs and presentation of neoepitopes to T-cells, which can trigger several immune responses. This first cascade of immune activation may cause further beta-cell stress or death, generating an autoreactive loop, with further modification of beta-cell proteins and disease exacerbation. This proposed model could provide evidence for a role of neoepitopes both in initiation and exacerbation of disease. It may indeed be that different ways of stress are needed to activate specific types of PTMs, at different times and with unknown duration, during the disease course. **(B)** Proposed roadmap to identification and utility of neoepitopes in T1D. Multiple neoepitopes have been identified in T1D and yet their precise role in the disease remains ambiguous. We envision two major areas of research: 1) to improve the identification of neoepitopes and to better delineate their role in disease pathogenesis; 2) to evaluate their use as biomarkers and their therapeutic utility.

These environmental insults may lead to the formation of a first wave of non-conventional proteins. Impaired clearance of such stressed or dying beta-cells expressing modified proteins will cause activation of APCs and presentation of neoepitopes to CD4+ T-cells, which in turn can trigger several immune responses, including B cell activation with autoantibody production, and the activation of antigen-specific effector T cells that can directly kill beta-cells presenting modified islet peptides. This first cascade of immune activation may in this way cause further beta-cell stress or death, generating an autoreactive loop, with further modification of beta-cell proteins and disease exacerbation. Moreover, epitope spreading to native epitopes may further amplify the immune response (Yang et al, submitted). This proposed model could provide evidence for a role of neoepitopes both in initiation and exacerbation of disease (**Figure 2A**). It may indeed be that different ways of stress are needed to activate specific types of PTMs, at different times and with unknown duration, during the disease course.

## Where Do We Go From Here?

To get a full picture of the neoepitope landscape, more studies should be carried out evaluating such responses in the early stages of T1D and through their progression to T1D to further delineate and describe the importance and timing of neoepitope formation in T1D. Elucidating their role in pathogenesis may also enable their use to improve the current staging paradigm, which is largely based on the presence of autoantibodies to classical islet antigens. Finally, while antigen-specific approaches have shown limited efficacy to date in T1D prevention and treatment, it is feasible that targeting neoepitopes in addition to or instead of classic antigens may provide a better therapeutic benefit.

Understanding how neoantigens are involved in the break of tolerance will lead us towards tolerogenic therapies for T1D. Multiple antigens have been used in T1D trials (83, 84). While no trial achieved its primary outcome, two trials have shown partial successes. First, in a trial where oral insulin was administered to individuals with stage 1 T1D, a pre-defined subgroup had a significantly delayed time to diabetes development (85). Furthermore, in a trial with intranodal injection of GAD65, a predefined subgroup showed preservation of beta-cell function (https://www.diamyd.com/docs/pressClips.aspx?ClipID=3768129). Other groups are exploring combination of antigens and/or antigens plus immunomodulators. Given the limited success of single native antigen in inducing tolerance, the presence of epitope spreading in T1D and the evidence for neoantigen generation and autoreactivity, it is quite feasible that targeting multiple (neo)antigens could aid in promoting antigenic tolerance.

Based on current evidence, we wish to outline two major areas of research to better delineate their exact role, which is a prerequisite for their therapeutic utility (**Figure 2B**). First, to identify novel epitopes that are most relevant to T1D progression, it will be necessary to develop and use novel detection and analysis methods with improved sensitivity and capacity to identify the nature of the peptides that are presented (86). In turn, being able to assay for T-cell reactivity, even in the periphery, remains a major challenge. The identification of novel epitopes could open opportunities for the characterization of disease subtypes and broaden our understanding of the disease pathogenesis. More efforts on the assessment of circulating antibodies and T-cell reactivity in longitudinal samples are needed to fully understand the timing and nature of neoepitope autoreactivity. Although sample volume continues to be a limitation, especially in studies of pediatric population, we anticipate that new technologies will be able to significantly improve our capacity to detect autoreactive cells in these challenging samples.

It is conceivable that neoepitopes may be used alone or in conjugation with other antigens as biomarkers for disease severity and/or progression, with evident potential to become immune-modifying therapies to induce tolerance in T1D. We could imagine a scenario in which each diabetic patient has its own antigenic profile, which could be used towards personalized medicine. However, more robust preclinical models or *in vitro* systems to test antigenic candidates should be developed prior to entering clinical studies. In all, neoepitopes possess a yet untapped mechanism to provide better biomarkers for staging progression as well as therapeutic targets, and could be the key to understanding the loss of tolerance in T1D.

## Data Availability Statement

The original contributions presented in the study are included in the article. Further inquiries can be directed to the corresponding author.

## Author Contributions

TR-C, JDJ, LO and JLD conceived the concept and co-wrote the manuscript. All authors contributed to the article and approved the submitted version.

## Funding

Research in this area in the JJ lab is supported by Diabetes Canada and CIHR. Related research in the LO and TR-C labs is supported by IMI2-JU under grant agreement No 115797 (INNODIA) and No 945268 (INNODIA HARVEST). This Joint Undertaking receives support from the Union’s Horizon 2020 research and innovation program and “EFPIA”, ‘JDRF” and “The Leona M. and Harry B. Helmsley Charitable Trust”. LO lab is supported by JDRF (1-SRA-2019-809-S-B). TR-C is supported by JDRF (5-CDA-2020-949-A-N).

## Conflict of Interest

JLD is an employee of Janssen Research and Development, LLC.

The remaining authors declare that the research was conducted in the absence of any commercial or financial relationships that could be construed as a potential conflict of interest.
